# From Bacteria to Piscivorous Fish: Estimates of Whole-Lake and Component-Specific Metabolism with an Ecosystem Approach

**DOI:** 10.1371/journal.pone.0101845

**Published:** 2014-07-11

**Authors:** Fabien Cremona, Toomas Kõiv, Veljo Kisand, Alo Laas, Priit Zingel, Helen Agasild, Tõnu Feldmann, Ain Järvalt, Peeter Nõges, Tiina Nõges

**Affiliations:** 1 Estonian University of Life Sciences, Institute of Agricultural and Environmental Sciences, Tartu, Estonia; 2 University of Tartu, Faculty of Science and Technology, Institute of Technology, Tartu, Estonia; Dauphin Island Sea Lab, United States of America

## Abstract

The influence of functional group specific production and respiration patterns on a lake's metabolic balance remains poorly investigated to date compared to whole-system estimates of metabolism. We employed a summed component ecosystem approach for assessing lake-wide and functional group-specific metabolism (gross primary production (GPP) and respiration (R)) in shallow and eutrophic Lake Võrtsjärv in central Estonia during three years. Eleven functional groups were considered: piscivorous and benthivorous fish; phyto-, bacterio-, proto- and metazooplankton; benthic macroinvertebrates, bacteria and ciliates; macrophytes and their associated epiphytes. Metabolism of these groups was assessed by allometric equations coupled with daily records of temperature and hydrology of the lake and measurements of food web functional groups biomass. Results revealed that heterotrophy dominated most of the year, with a short autotrophic period observed in late spring. Most of the metabolism of the lake could be attributed to planktonic functional groups, with phytoplankton contributing the highest share (90% of GPP and 43% of R). A surge of protozooplankton and bacterioplankton populations forming the microbial loop caused the shift from auto- to heterotrophy in midsummer. Conversely, the benthic functional groups had overall a very small contribution to lake metabolism. We validated our ecosystem approach by comparing the GPP and R with those calculated from O_2_ measurements in the lake. Our findings are also in line with earlier productivity studies made with ^14^C or chlorophyll *a* (chl-*a*) based equations. Ideally, the ecosystem approach should be combined with diel O_2_ approach for investigating critical periods of metabolism shifts caused by dynamics in food-web processes.

## Introduction

The main processes governing aquatic ecosystem metabolism are primary production and respiration. Gross primary production (GPP) is the fixation of inorganic carbon (C) by autotrophs while community respiration (R) is the remineralisation of organic C to CO_2_ by all organisms of the ecosystem [1]. The net ecosystem production (NEP) showing the difference between GPP and R, (NEP  =  GPP - R), can be used to define the metabolic type (net auto- or heterotrophic) of an ecosystem [2]. Lakes constitute suitable subjects for whole-ecosystem studies because they have clear ecosystem boundaries [3], but it is still unclear which factors determine whether they are autotrophic or heterotrophic on an annual basis.

For assessing the metabolism of lakes, the most popular approach in the last decades has been calculating GPP and R from measured integrated CO_2_ and/or O_2_ fluxes in the surface waters ([4], [5], [6]). These fluxes are supposed to represent the contribution of primary production and respiration of photoautotrophic functional groups (phytoplankton, phototrophic bacteria, periphyton and macrophytes) and respiration of heterotrophic functional groups (zooplankton, zoobenthos, heterotrophic bacteria, fish) to the whole NEP.

However, the free-water gas exchange approach has recently been questioned due to its inability to discern the drivers of lake ecosystem metabolism ([7]) and to partition the GPP and R component rates ([6]). Especially, the seasonal contribution of different functional groups to the overall net autotrophy or heterotrophy of the system cannot be assessed using this method only. The use of the CO_2_ based technique becomes problematic in alkaline (pH>8) lakes where the released CO_2_ is partly converted to bicarbonate and carbonate ions ([8]) and could thus be underestimated. Therefore, measuring community R and GPP solely is not suitable for tracing functional group specific production and respiration patterns and their influence on whole-lake metabolic balance.

The summed component ecosystem approach (hereafter “ecosystem approach”, [9]) is necessary for addressing these shortcomings. This method enables partitioning the metabolism into component-specific parts and offers also a robust estimate of whole-lake metabolism, especially if combined with in-lake short-term rate measurements ([10]). However, the ecosystem approach requires detailed information about biomass of various functional groups in a lake [9] and, ideally, a good temporal resolution. For these reasons, this approach has been seldom used, and even then a fine temporal resolution of groups' metabolic rates has been often missing, so that only steady state models unable to depict the seasonal dynamics of functional group contribution to lake metabolism could be developed ([11]).

The large eutrophic hemiboreal Lake Võrtsjärv (Estonia) constitutes an ideal subject of a dynamic ecosystem approach for three reasons. Firstly, biomasses of different functional groups have been measured monthly in this lake since the 1960s and extensive metabolism studies have been carried out since 2009. Secondly, despite its large surface area (270 km^2^), Võrtsjärv is a very homogeneous system with a continuously mixed water column and little to no differences in temperature, concentrations of nutrients, and oxygen between lake zones ([12]). Thirdly, metazooplankton is able to graze only a small fraction of the organic matter produced by phytoplankton in this lake giving a particular importance to the microbial loop in mineralization processes ([13], [14]). Microbes have higher specific metabolic rates (i.e. they decompose more organic matter to CO_2_) than metazoans, implying that lakes dominated by microbial loop consumers should thus be more heterotrophic than lakes where metazoans are the main consumers ([15]).

Our objective was to compare the whole-lake and functional group specific carbon metabolism in Võrtsjärv. We employed an original method based on biomasses and allometric equations for food web functional groups coupled with daily records of water temperature and hydrology. The following functional groups were considered: piscivorous and benthivorous fish; phyto-, bacterio-, proto- and metazooplankton; benthic bacteria, ciliates and invertebrates, macrophytes and their associated epiphytes. Our working hypothesis was that phytoplankton and microbial loop consumers (protozooplankton and heterotrophic bacterioplankton) make up the majority of lake metabolism.

## Methods

### Site description

Võrtsjärv is a large (270 km^2^), eutrophic (average concentrations of chl-*a* 31 mg m^−3^, total phosphorus 50 mg m^−3^, total nitrogen 1400 mg m^−3^) lake located in Central Estonia (58°05′–58°25′ N and 25°55′–26°10′ E). The catchment area of the lake is 3104 km^2^ of which 40% (1313 km^2^) is used for agriculture, followed by agricultural drained lands (799 km^2^, 26.6%), forest (749 km^2^, 24%), and bogs (243 km^2^, 8%). The lake itself is shallow, with mean depth of 2.8 m and maximum depth 6 m. The water is alkaline (pH 7.5–9). Due to its shallowness and large wind-exposed area, Võrtsjärv is mixed and highly turbid. Water transparency by Secchi depth does not exceed 1 m during the ice-free period. Water-level fluctuations are considerable with an average amplitude of 1.4 m and a maximum range of 3.2 m ([16]). Community composition of plankton, fish and macrophytes can be found in the references provided in corresponding sub-sections and in tables S1 and S2.

### Study design

We chose an ecosystem approach ([17]) for determining Võrtsjärv net ecosystem productivity which can be summarized by the following equation:
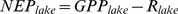
with GPP_lake_ and R_lake_ being, respectively, whole-lake gross primary production and biological mineralization of carbon (total respiration). Positive NEP_lake_ values (GPP_lake_ > R_lake_) indicate that the lake is autotrophic, negative NEP_lake_ values (GPP_lake_ < R_lake_) denote that the lake is heterotrophic and decomposes more organic matter than it produces. Benthic primary production (PP) by periphyton can be considered negligible in Võrtsjärv because the compensation point (<2 m, [18]) lies higher than mean depth (2.8 m). In Võrtsjärv, the majority of PP is made by planktonic cyanobacteria ([19], [20]). Diatoms are also present but mostly as planktonic species and might dominate during very short periods just after ice-off. Thus epipelic and epipsammic PP are assumed to be very low as it is generally observed in turbid lakes [21]. Respiration by planktivorous fish can also be omitted because this functional group always represents a tiny (<1.5%) fraction of fish biomass in Võrtsjärv [22]). Emergent and floating-leaved macrophytes metabolism was not taken into account for lake carbon metabolism assessment for two reasons: (I) most of the assimilatory and respiratory CO_2_ exchange of emergent and floating-leaved vascular plants is directly with the atmosphere, and most of plant remains are decomposed in the beach ridge; (II) the uncertainty of the contribution of emergent macrophytes to lake metabolism is further increased because of water level changes in Võrtsjärv leaving large littoral areas temporarily dry and isolated from the water. Thus, only phytoplankton, submerged macrophytes and their associated epiphytes were considered in primary production calculations. For calculating respiration, we took into account all planktonic components (bacterioplankton, phytoplankton, protozooplankton, and metazooplankton), benthic macroinvertebrates, ciliates and bacteria, and also benthivorous and piscivorous fish. Epiphytes were included together with macrophytes to respiration calculations because models generally do not display separate epiphyte respiration rates [23]. GPP_lake_ and R_lake_ can thus be partitioned among the following main functional groups:
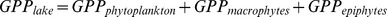
and
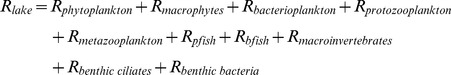
where R_pfish_ and R_bfish_ represent respiration by piscivorous and benthivorous fish, respectively. GPP_macrophytes_ and R_macrophytes_ are the primary production and respiration of submerged vascular plants.

Staehr and Sand-Jensen [5] observed that daily assessments of lake metabolism provide the optimal background for evaluating temporal changes accurately enough. Since most hydrology and temperature data for Võrtsjärv were measured on a daily basis during the ice-free period, we chose a daily increment for calculations, with a total duration of 1095 days (i.e. three complete non leap years) from the 1^st^ of January 2009 to the 31^st^ of December 2011. This means that for each day, the values of all the equation members described previously would be entered into the equations (2) and (3) so that functional group specific respiration and primary production, GPP_lake_ and R_lake_ can be assessed. For the variables that could not be measured daily, an average monthly value was used instead.

### Measurements of lake physical parameters

Daily water level (WL_abs_, m.a.s.l) data measured at the outlet of the lake was obtained from the Estonian Institute of Hydrology and Meteorology (EMHI). The mean depth of the lake (Z_avg_), water volume, and surface area were calculated from their relationships to the water level as given by [24] and [25]. As lake temperature (°C) measurements are described precisely in Laas et al. [26], the methods are only shortly summarized below. From January 2009 to December 2011, temperature was measured using a multiparameter probe (YSI6600 V2–4). All measurements were done at 1-m depth with 10–15 min intervals, and were then hourly averaged.

### Sampling of functional groups

No specific permission is required for accessing and conducting research in an Estonian public water body.

#### Plankton

Unless specified otherwise, water samples were collected in 2009–2011 at monthly interval from the monitoring station near the eastern shore where the water depth corresponded to the mean lake depth. Depth integrated lake water was gathered over the whole water column with a Ruttner sampler, mixed in a 50-L jar and sampled for plankton. For bacterial abundance, 20-mL samples were preserved with 4% (final concentration) formaldehyde. Each sample was filtered onto a black 0.22 µm pore-size polycarbonate filter (Osmonics Inc.) and stored at -21°C until the analysis. Bacterial abundance was determined by epifluorescence microscoping (Leica DM RB) at ×1000 magnification after staining with DAPI (4′,6′-diamidino-2-phenylindole; PolySciences Inc.) at final concentration of 10 µg mL^−1^. Bacterial cells in 20 fields were counted (always n>200 cells). At the microscope 50 cells (or more to achieve S.E.<20%) per field were sized visually by comparison to the globes of a calibration eyepiece graticule (Patterson Globe and Circle, GI, Eyepiece Graticules Ltd.). Biomass was calculated from the average biovolume of bacterial cells using conversion factors from biovolume to carbon biomass (380 fg C µm^−3^ [27]).

For the other planktonic functional groups, 100-mL samples (from 10 L first filtrated through 48 µm mesh for metazooplankton) were fixed with acid Lugol's iodine solution [0.5% (vol/vol) final concentration] and identified at the species level. Phyto- and protozooplankton composition and biomass were analysed using Utermöhl's technique ([28]). Cells were enumerated with an inverted microscope at ×400 magnification. The samples were counted until reaching at least 400 counting units (filaments, cells, colonies) for phytoplankton (which gives a counting error of ±10% for the total biomass) or the first 20 specimens encountered for each taxon (protozooplankton). Biovolumes of taxa were estimated by assuming simple geometric shapes. Specific gravity of 1.0 g ml^−1^ was assumed [29] and the biomass was expressed as wet mass. Protozooplankton wet weight (µg L^−1^) was then converted into carbon biomass (mg C m^−3^) by using a conversion factor of 1 g WW  = 0.071 g C [30].

Metazooplankton was counted under a dissecting microscope in a Bogorov chamber and enumerated at 60× magnification. Individual wet weights of rotifers were estimated from average lengths, according to Ruttner-Kolisko [31]. The lengths of crustaceans were converted to wet weights, according to Studenikina and Cherepakhina [32] for nauplii and Balushkina and Winberg [33] for other groups.

#### Benthos

Bacterial abundance and biomass were measured as described in Tšertova et al. [34], [35]. Sediment cores containing 20 cm thick superficial sediment layer together with 10 cm thick water layer above it were taken with a Willner-type sediment gravity corer and sliced from top down into 2 layers bordered with 0–0.5 cm and 0.5–1 cm sediment depth. Bacterial abundance was determined in 0.5 ml of sediment fixed in glutaraldehyde (final conc. 1%). Prior to counting, the sample was mixed with 2.9 ml filtered (pore size 0.2 µm) lake water, and 0.6 ml sodium pyrophosphate (500 mM) was added [36]. Thereafter the samples were sonicated for 5 min at 4°C (Bandelin Sonorex Digital 10P, 480W). Gravity sedimented samples were diluted in DMSO at a volumetric ratio of 1∶20, stained with SybrGreen I (final concentration 5 µM). Particle counting was done on flowcytometer (BD LSR II, exitation by Solid state Sapphire L1 488 nm, band pass filters 530/30 nm). Abundance was converted to biomass using average carbon content (20 fg C per cell). Data were normalized to sediment dry weight (cells g^−1^ DW).

Samples for benthic ciliate analysis were collected seasonally between August 2005 and May 2006 using a core sampler. Only the topmost layer (1 cm) was analysed. Samples were fixed with glutaraldehyde (final concentration 1%). Ciliates in the top 1 cm of core samples were enumerated using Utermöhl [28] technique. Samples were diluted to a level at which all ciliate species were easily discriminated from sediment and detritus particles. Samples were analysed using inverted microscope Zeiss Axiovert 100 (400–600x); the whole counting chamber was surveyed. The conversion factor from biovolume to carbon biomass was 190 fg C mm^−3^.

Invertebrate samples were collected monthly in the benthic zone of the lake with a Boruckij-type sampler (a modification of the Ekman grab, grasp area 225 cm^2^, box height 40 cm). Five replicates from the deepest part of the lake were taken each month. Macrozoobenthos samples were sieved (mesh size 0.3 mm) in situ, sorted alive by eye in laboratory and then fixed in 70% ethanol. After the removal of the exterior moisture on blotting paper, wet biomass of ethanol-fixed animals was estimated on torsion weights (with an accuracy of 1 mg). When possible, the species of animals were identified while, in most cases a higher identification level than genus was used.

#### Fish

Fish biomass was assessed during fishing campaigns which took place during ice-free periods of autumn 2009–2011 (12 in 2009 and 2011, 11 in 2010). Fishing campaign was authorized by the Ministry of Environment in Estonia (permit numbers: 892706, 959220 and 1036478 for 2009, 2010 and 2011 respectively) and conducted under its guidelines. A bottom trawl (height 2 m, width 12 m, 10–12 mm knot-to-knot mesh size at the cod-end) was employed and towed by a ship for 30 min per haul at a speed of 5.5–6.2 km h^−1^. Sampling was conducted at midday in the pelagic zone of the lake at 5 different sites covering a cumulative surface of 5.5 ha. Caught fish were immediately killed by stunning in order to minimize suffering and stress. This method is approved by Ministry of Environment in Estonia. Fish were classified into feeding groups according to their main adult feeding mode, literature data, and lake specific preferences (Järvalt et al. 2004). Pike (*Esox lucius* L.), pikeperch (*Sander lucioperca* (L.)), perch (*Perca fluviatilis* L.) and burbot (*Lota lota* (L.)) were considered piscivorous, whereas eel (*Anguilla anguilla* (L.)), roach (*Rutilus rutilus* (L.)), bream (*Abramis brama* (L.)), white bream (*Blicca bjoerkna*) and ruffe (*Acerina cernua* (L.)) benthivorous species. Benthivorous and piscivorous fish species represented between 95 and 99.5% of catches [22].

### Calculation of metabolism

Primary production was calculated by methods specified for each primary producer in sections below. For calculating the respiration of unicellular organisms, we used mostly allometric equations, while wet weight taxa-specific relationships were used for multicellular organisms. When necessary, temperature-correction was made to the regressions, assuming a coefficient (Q_10_) of 1.38 for planktonic algae [37], 1.6 for bacteria [38], 2 for protozoans [30], and 2.25 for metazooplankton [39]. For regressions which yielded results in O_2_ units, the conversion to carbon units was made assuming the gas molar volume of 22.4 L at 1 atm pressure and using a respiratory quotient (RQ) of 1 for plants [40], zooplankton [41] and bacteria [42]. All volume- and surface-specific respiratory C values were then extrapolated, correspondingly, to the lake volume (planktonic functional groups) or surface (benthic functional groups, macrophytes and epiphytes).

#### Phytoplankton

Phytoplankton primary production (GPP_phytoplankton_) was estimated using an integral version of the semi-empirical model elaborated by Arst et al. [43]. Chlorophyll a concentration (96% ethanol extract) was analysed spectrophotometrically and calculated according to Lorenzen [44]. The model used as input variables monthly measured chl-*a*, hourly incoming irradiance, and daily diffuse attenuation coefficient. We assumed that no production was taking place when the lake was ice-covered. Smoothing and corrections for modelled results were made as described in Nõges et al. [45]. For respiration (R_phytoplankton_) we multiplied cell-specific respiration rates by the number of cells of each species. For filamentous species, the number of cells was obtained by dividing filament length by mean cell length. For colonial species like *Microcystis* sp., we used Joung et al. [46] method: 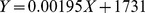
where Y is the number of cells in a colony and X is the volume of the colony (10^5^ µm^3^).

For taxa whose cell-specific respiration rates were documented in the literature, we used those (Table 1). If respiration rates were unavailable for a taxon, an allometric regression described in Tang and Peters [37] was employed instead:

**Table 1:** Methods used for assessing phytoplankton respiration and cell size.

Taxon	Cell respiration value (pg C day^−1^)	References	Cell size estimation	References
*Aphanizomenon* spp.	2.3	[72]	-	-
*Limnothrix* spp.	0.13	[73]	-	-
*Oscillatoria* spp.	0.13	[73]	-	-
*Planktolyngbya* spp.	0.13	[73]	-	-
*Microcystis* spp.	2.16	[74]	Based on colony volume	[46]
Other phytoplankton taxa*	R = 0.0068 V ^0.88^	[37]	Based on size class	[47]

*R: respiration rate (pl O_2_ cell^−1^ h^−1^); V: cell volume (µm^3^). R was then converted into carbon units using a respiration quotient of 1 and a molar volume of 22.4 L at 1 atm.



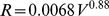



Where the respiration (R, pL O_2_ cell^−1^ h^−1^) is a function of cell volume (V, µm^3^). This regression was chosen because it is valid across several orders of magnitude of cell sizes which is the case in phytoplankton communities. Cell volume was calculated for each taxon according to the Nordic algae database [47].

#### Macrophytes and epiphytes

Sampling and measurement methods are described more thoroughly in Nõges et al. [48] but are briefly summed up here. Hourly primary production of submerged macrophytes and their associated epiphytes was measured *in situ* in summer 2008 with the ^14^C uptake method on *Myriophillum spicatum* L. which is the most abundant submerged macrophyte species in Võrtsjärv. Pieces of leaves from upper, middle and lower sections were cleaned from epiphytes by vigorous shaking in a vessel filled with water during 2 min. Hourly PP was converted to daily PP (mg C g^−1^ day^−1^) using the following equation from Nõges and Nõges [49]:
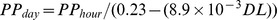



Were DL is the number of hours of daylight. Both macrophytes' and epiphytes' PP (GPP_macrophytes_, GPP_epiphytes_) were expressed from dry weight biomass of macrophytes in August or September. For winter months (January to March) we assumed that macrophyte biomass was 50% of that in September [23]. Values of other months were obtained by exponential fitting of these measured data [23]. The estimated area covered by submerged macrophytes (15%, [48]) was multiplied by the PP_day_ for assessing whole lake GPP_macrophytes_ and GPP_epiphytes_.

Respiration of submerged macrophytes (R_macrophytes_) and their associated epiphytes (g O_2_ g DW^−1^ day^−1^) was calculated with the following biomass and temperature dependent regression used by Plus et al. [23]:
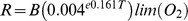



Where B is the macrophyte biomass (g DW m^−2^), T is daily average water temperature (°C), and lim (O_2_) – a limitation function for the macrophyte respiration since respiration rates of primary producers decrease with the concentration of dissolved oxygen:
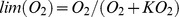



KO_2_ is the half-saturation coefficient whose value is fixed at 5 gO_2_ m^−3^. Dissolved O_2_ data for 2009 to 2011 was obtained from high-frequency measurements (see [26] for the measurement procedures).

#### Planktonic and benthic consumers

The metazooplankton respiration (R_metazooplankton_; ml O_2_ h^−1^ g^−1^) was calculated on the basis of relationship with wet weight (W, g):


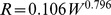
 for Rotifera [50]


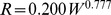
 for Copepoda [51]


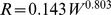
 for Cladocera [51]

Protozooplankton and benthic ciliates respiration (R_protozooplankton_, R_benthic ciliates_; ml O_2_ ind^−1^ h^−1^) was assessed using Fenchel and Finlay [30] equation:
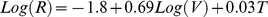



Where V stands for individual cell volume (1 µm^3^  = 1 pg WW  = 10^−6^ µg WW). Only one third of the lake sediment surface offers suitable area for benthic ciliate colonization. In the other parts of the profundal zone the substratum is too compacted [52]. We thus assumed a colonization depth of 2 cm for 1/3 of the lake and 0.5 cm only for the remaining 2/3 averaging a lake-wide depth of 1 cm that is suitable for benthic ciliates.

For bacterioplankton and benthic bacteria respiration (R_bacterioplankton_, R_benthic bacteria_) we did not use bacterial growth efficiency because it is too unpredictable for being a reliable proxy for respiration, ranging from <0.05 to 0.6 [53]. Bacterial respiration was instead calculated with the cell-carbon-content dependent equation modified from Tang and Peters [37] and multiplied by bacterial abundance:
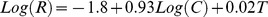



Where R is the respiration rate (pL O_2_ cell^−1^ h^−1^) and C is the carbon content of a cell (pg C cell^−1^).

For macrozoobenthos, the three dominant macroinvertebrate taxa in Võrtsjärv, which represent the overwhelming biomass of the benthos biomass [54] were considered: Chironomidae, Oligochaeta, and Unionidae. Respiration of macroinvertebrates (R_macroinvertebrates_) was calculated with respiration rates for these taxa reported by Jónasson [55]. We chose to use respiration rates corresponding to air saturation conditions because Võrtsjärv water column is constantly mixed and seldom experiences hypoxia. These rates are 29.9, 13.1 and 16.8 µL O_2_ g ww^−1^ h^−1^ for Chironomidae, Oligochaeta and Unionidae, respectively.

#### Fish

Biomass of piscivorous and benthivorous fish obtained from the 2009–2011 annual results of fishing campaigns (see above and [22]) was converted into dry weight (DW) biomass (g DW m^−2^), carbon units (g C m^−2^) and respiration (R_bfish_, R_pfish_; g C m^−2^ d^−1^) using conversion factors of 0.2, 0.492 and 0.033 respectively, provided by Andersson and Kumblad [11].

### Data treatment and model validation

Missing data were interpolated with the closest monthly values. Before display, GPP and R data were transformed with exponential trend smoothing (StatSoft, Inc. (2011) STATISTICA data analysis software system, version 10 www.statsoft.com). Validation of the model was done with two different methods. Firstly, the calculated GPP and R were plotted with GPP and R values calculated by Laas et al. [26] in the same lake with O_2_ fluxes method (Cole et al 2000) during the ice-free period. Secondly, planktonic respiration calculated with our method was compared with models of planktonic respiration from del Giorgio and Peters [40] and Duarte and Agusti [56]. For these two models R was predicted based on either chl-*a* [40] or GPP [56].

## Results

### Biomasses of planktonic functional groups

**Figure 1:**
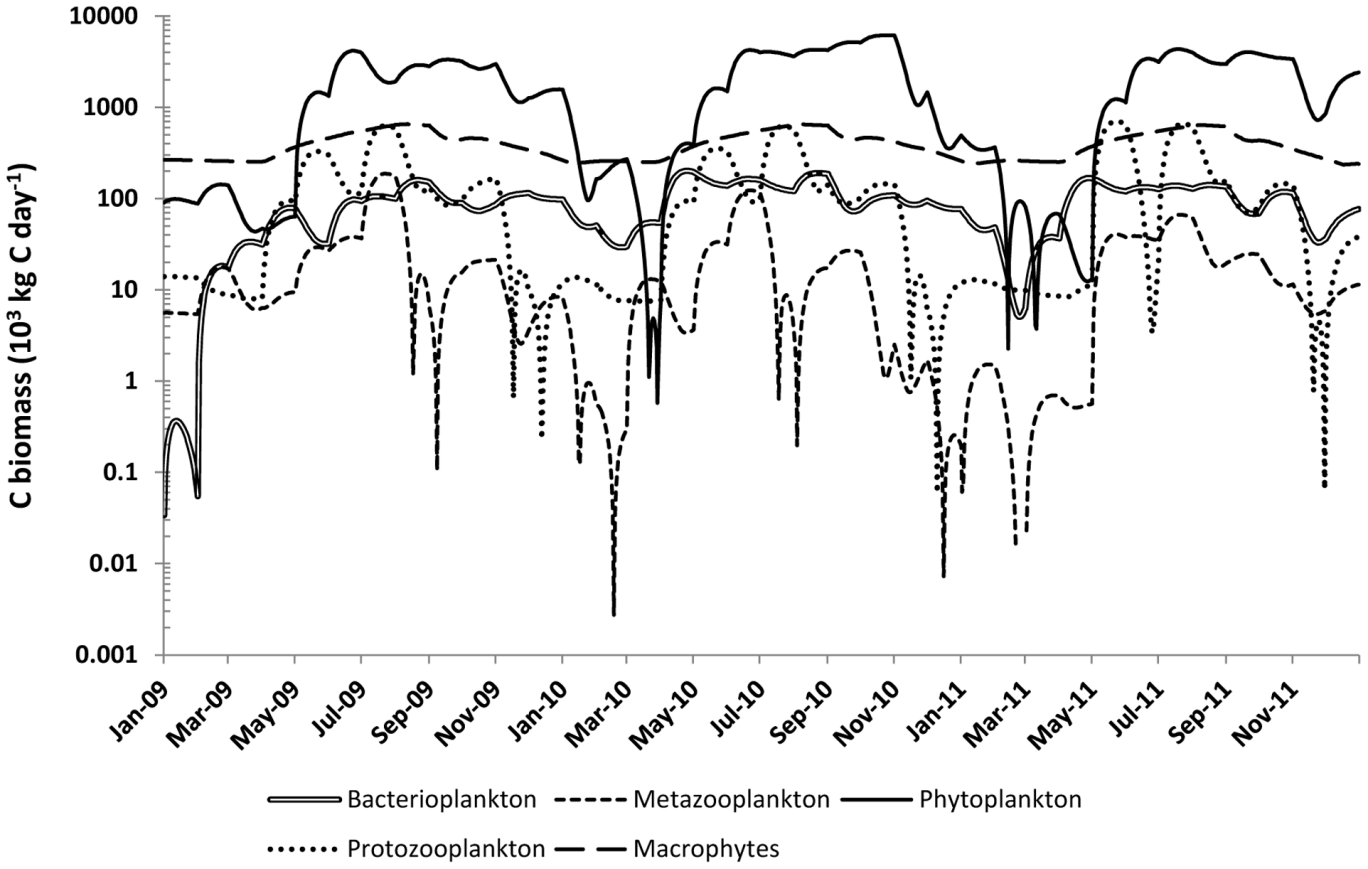
Dynamics of lake-wide functional groups biomass (10^3^ kg day^−1^) during the 2009–2011 period Biomass values are shown on a logarithm scale for clarity purposes.

Lake-wide planktonic group biomasses exhibited seasonal variation across five orders of magnitude (Fig. 1, Table 2). Biomasses were low in the early spring and high during the rest of the ice-free season. Phytoplankton built up by far the largest average biomass (1.9 10^6^ kg C lake-wide). The organisms of the microbial loop, protozooplankton and bacterioplankton, represented the largest groups of consumers with 137 and 90 10^3^ kg C respectively. Biomass variation was huge on a seasonal basis, often spanning three orders of magnitude for protozooplankton. Metazooplankton biomass was the lowest among planktonic functional groups, with an average of only 20 10^3^ C lake-wide.

**Table 2:** Average calculated biomass, primary production and respiration of Lake Võrtsjärv functional groups during the 2009–2011 period (n = 1095).

Functional groups	Biomass	Biomass	GPP	GPP	R	R	R/R_lake_
***Plankton***	*10^3^ kg C*	*g C m^−3^*	*10^3^ kg C day^−1^*	*mg C m^−3^ day^−1^*	*10^3^ kg C day^−1^*	*mg C m^−3^ day^−1^*	*(%)*
Phytoplankton	1 932 (14–6048)	3 (0.01–8)	164 (0–618)	206 (0–715)	128 (1–352)	168 (0–440)	43 (3–84)
Bacterioplankton	90 (0–187)	0.1 (0.001–0.24)			42 (0–117)	52 (0–142)	20 (0–51)
Metazooplankton	20 (0–158)	0.03 (0.001–0.1)			7 (0–50)	8 (0.01–59)	2 (0–10)
Protozooplankton	137 (8–565)	0.16 (0.09–0.7)			46 (1–324)	57 (1–410)	12 (1–50)
***Benthos and littoral***	*10^3^ kg C*	*g C m^−2^*	*10^3^ kg C day^−1^*	*mg C m^−2^ day^−1^*	*10^3^ kg C day^−1^*	*mg C m^−2^ day^−1^*	*(%)*
Macrophytes	400 (230–650)	10 (6–16)	16 (0–99)	400 (0–2300)	11 (0.5–88)	271 (11–2100)	3 (0–12)
Epiphytes*	-	-	0.04 (0–0.1)	1 (0–3)	-	-	-
Benthic macroinvertebrates	690 (22–3000)	2.5 (0.08–10.5)			1 (0–4)	4 (0.1–14)	0.8 (0–3.5)
Benthic ciliates	7 (2–14)	0.02 (0–0.05)			1 (0.5–2)	4 (2–8)	1 (0.1–4)
Benthic bacteria	6 (0.4–14)	0.02 (0.001–0.05)			2.5 (0.1–8)	9 (0.4–27)	1.4 (0–8)
***Fish***	*10^3^ kg C*	*g C m^−2^*			*10^3^ kg C day^−1^*	*mg C m^−2^ day^−1^*	*(%)*
Benthivorous fish	400 (3–6)	1.53 (1.1–2.3)			14 (10–23)	50 (38–76)	14 (1.2–62)
Piscivorous fish	100 (50–120)	0.3 (0.2–0.4)			3 (2–4)	10 (9–12)	3 (0–11)

Ranges are given in brackets.

*for respiration calculations macrophytes and epiphytes were considered together.

### Lake metabolism

**Figure 2:**
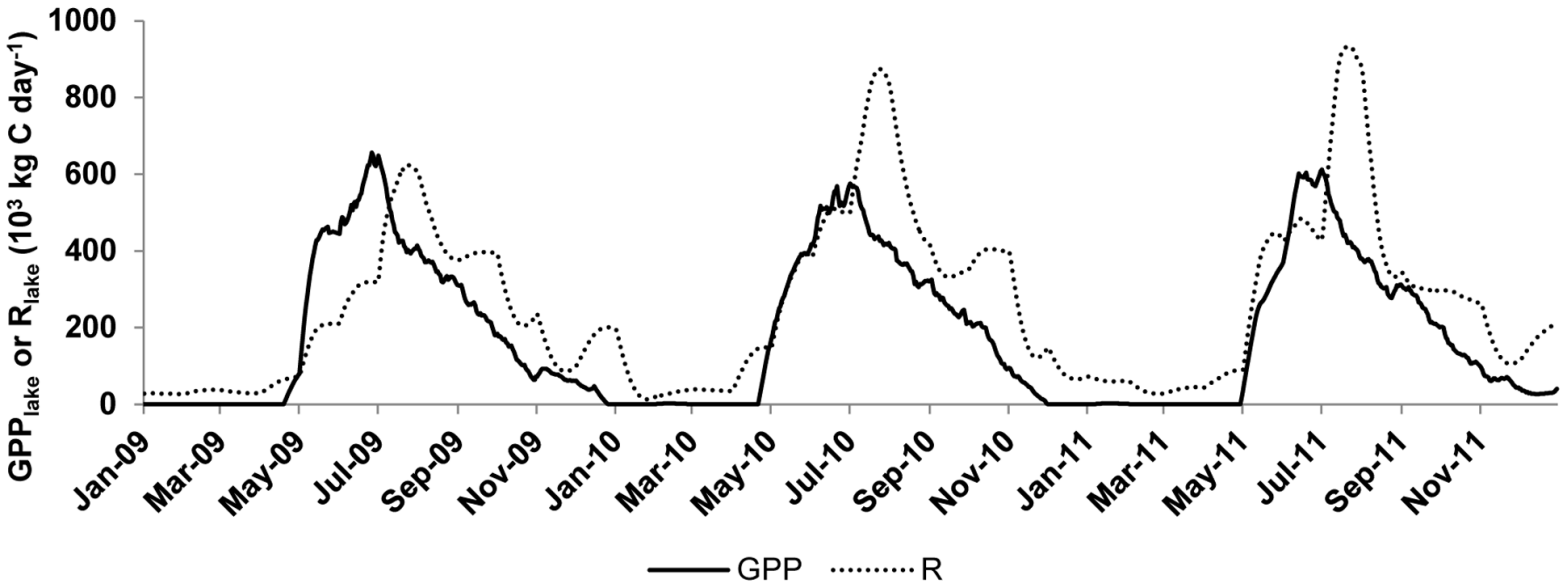
Dynamics of GPP_lake_ and R_lake_ during the 2009–2011 period calculated with ecosystem approach. Both parameters are expressed in 10^3^ kg C day^−1^.

**Figure 3:**
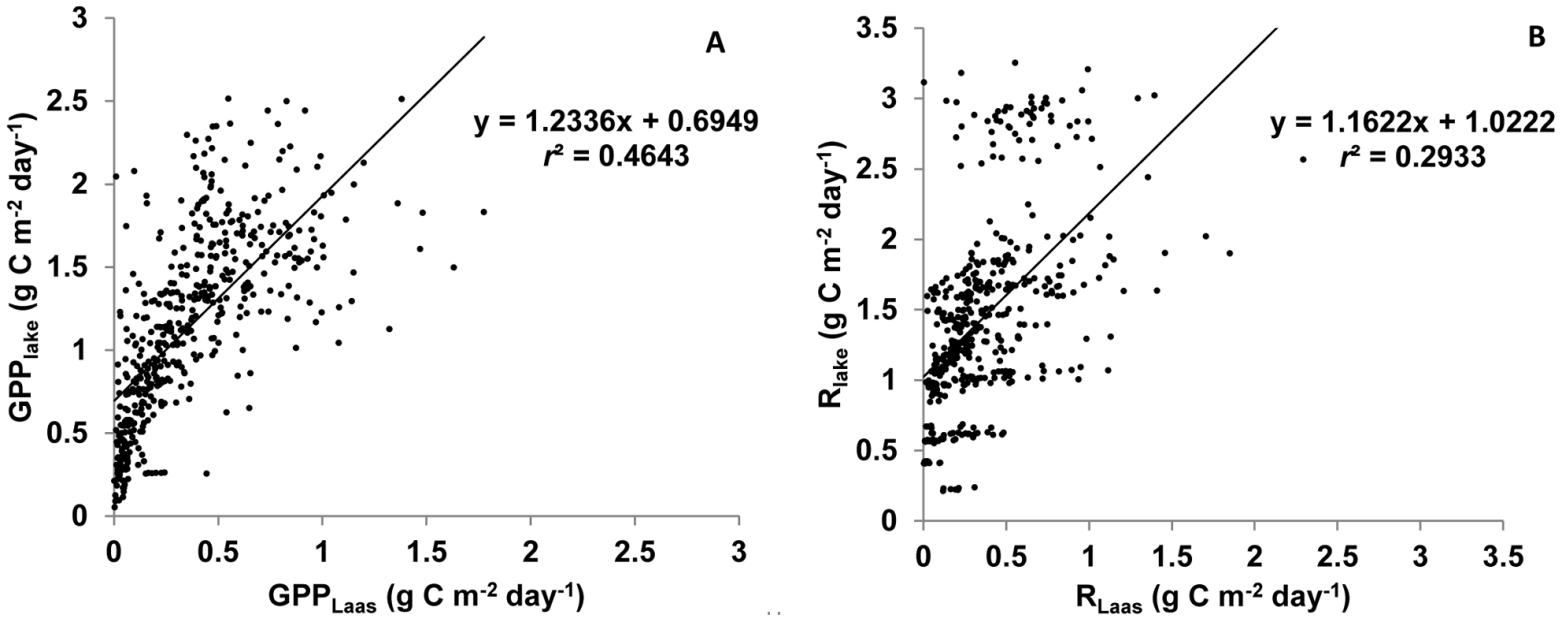
Scatter plot of GPP (A) and R (B) calculated in our study (GPP_lake_, R_lake_) and measured in Laas et al. [26] (GPP_Laas_, R_Laas_) from DO data during the ice-free seasons of 2009–2011 (n = 480). Parameters are expressed in g C m^−2^ day^−1^.

Võrtsjärv was generally heterotrophic, with annual average R_lake_ exceeding GPP_lake_ during the three years of study (Fig. 2). There was a clear seasonal pattern, wherein net autotrophy lasted only for a short period in late spring to early summer and corresponded to a stronger increase of GPP_lake_ relative to R_lake_ during this period. Yearly variations of metabolism were low compared to seasonal changes. GPP_lake_ slightly declined between 2009 and 2011 while respiration showed the reverse trend during the same period. The lake changed thus from weakly heterotrophic in 2009 to strongly heterotrophic in 2010 and 2011 mostly because of greater oxygen demand of protozooplankton. GPP and R calculated with the ecosystem approach were strongly correlated with GPP and R obtained by O_2_ fluxes method (Fig. 3). The relationship was especially strong for GPP (*R* ^2^ = 0.46), but showed a considerable coincidence also for R (*R* ^2^ = 0.29).

### Primary production and respiration of functional groups

**Figure 4:**
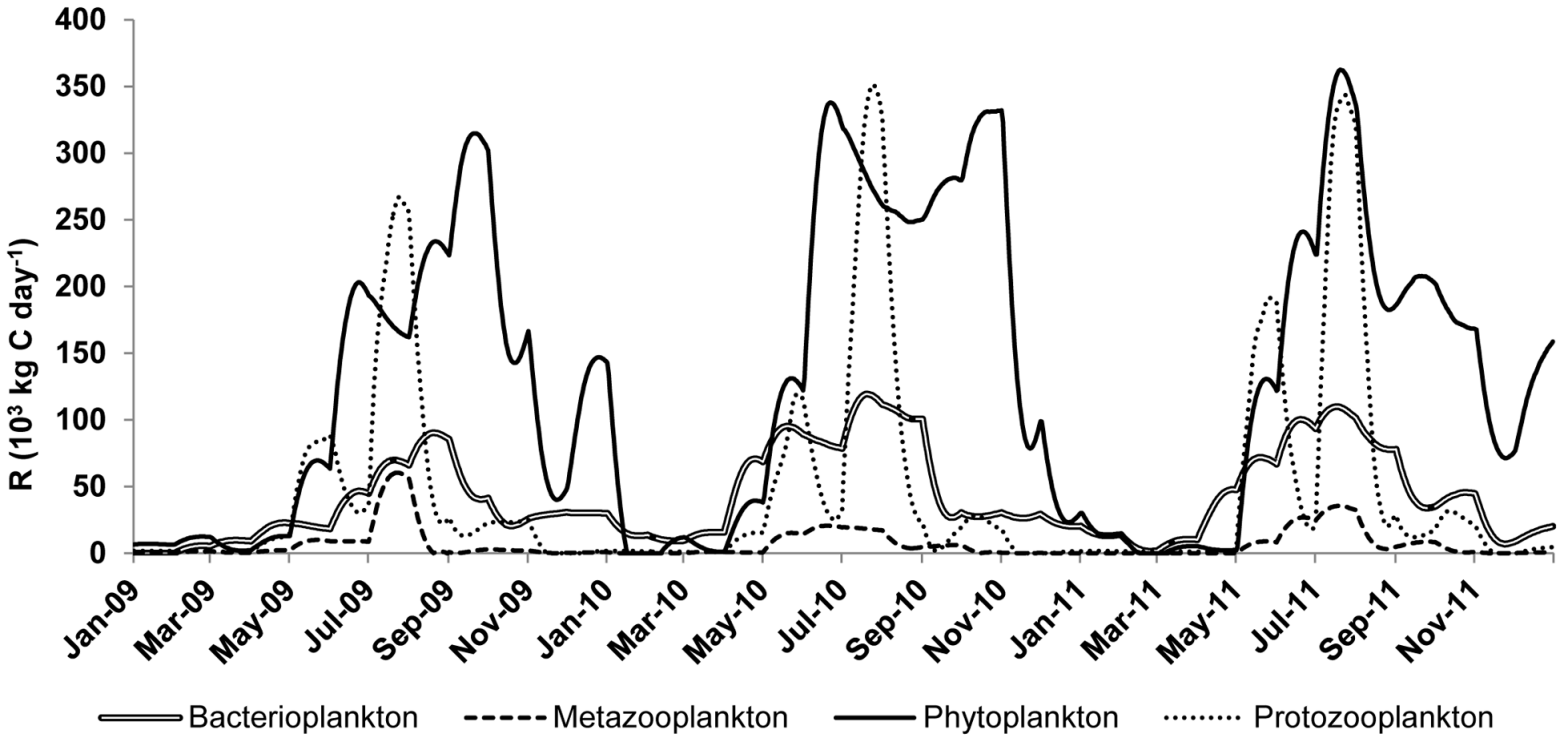
Calculated lake-wide respiration (10^3^ kg C day^−1^) of plankton functional groups during the 2009–2011 period.

The metabolic activity in Võrtsjärv was clearly dominated by planktonic functional groups (Table 2, Fig. 4). Organic matter was produced mainly by phytoplankton (GPP_phytoplankton_, avg. 164 10^3^ kg C day^−1^), followed by macrophytes (GPP_macrophytes_, avg. 16 10^3^ kg C day^−1^). The contribution of epiphytes was much lower (GPP_epiphytes_, 0.04 10^3^ kg C day^−1^; Table 2). Phytoplankton was also the dominant functional group in respiratory processes as R_Phytoplankton_ constituted on average 40% of R_lake_ and more than half of that of the plankton. Submerged macrophyte respiration was less than one-tenth of that of phytoplankton and accounted for 3% of whole-lake R. However, macrophytes had a greater GPP/R ratio than plankton (1.45 compared to 1.28) which indicates a greater relative net production of vascular plants.

**Figure 5:**
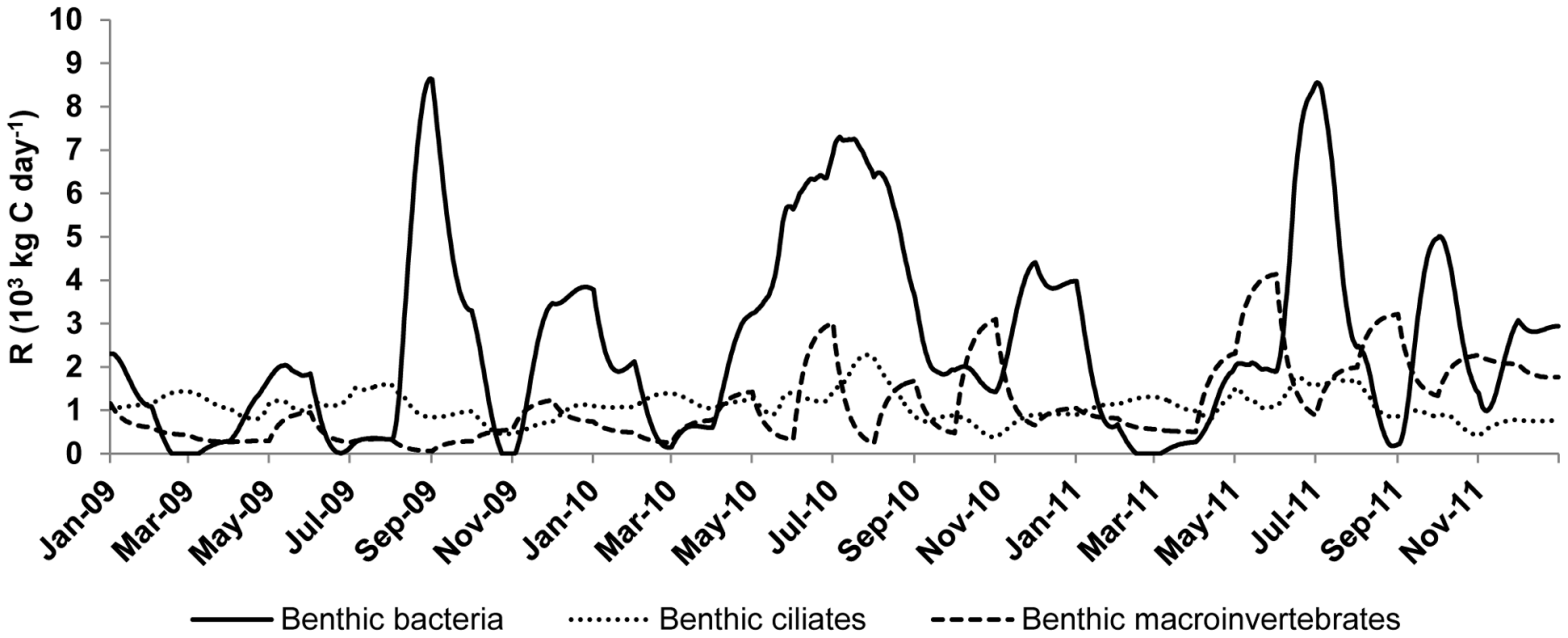
Calculated lake-wide respiration (10^3^ kg C day^−1^) of benthic functional groups during the 2009–2011 period.

Besides phytoplankton, the two most important functional groups in terms of contribution to R_lake_ were bacterio- and protozooplankton. The surge of protozooplankton and, to a lesser extent, bacterioplankton oxygen demand were responsible for bringing the lake back to heterotrophy after a short autotrophic summer period. Together these two functional groups respired 2/3 of the amount of C respired by phytoplankton. R_protozooplankton_ was coupled to its specific biomass and both variables fluctuated across three orders of magnitude from winter to midsummer. Conversely, metazooplankton contribution to metabolism of the lake was small (<2%) owing to its low overall biomass. Also the respiration of benthic functional groups was very low both in absolute and relative terms (Fig. 5). Macroinvertebrate and ciliate contributions were negligible (1%) on the whole-lake basis, with bacteria being the only benthic functional group to contribute up to 8% of the whole-lake metabolism. The share of fish was approximately 20% of the CO_2_ respired in Võrtsjärv, with the benthivorous fish contributing four times more than piscivorous fish.

## Discussion

### A heterotrophic plankton-dominated lake

During the three years studied, we found a clear seasonal pattern in Võrtsjärv metabolism. The lake was autotrophic during a short period in late spring 2009 (≈60 days) and 2011 (≈25 days) and heterotrophic the rest of the time. These findings are supported by those of Laas et al. [26] who reported a similar seasonal switching between autotrophy and heterotrophy although their results were based on water-column DO measurements. However, we have calculated that Võrtsjärv was net heterotrophic on a yearly basis whereas Laas et al. [26] considered it was weakly net autotrophic. Seasonal unbalance of measurements (no winter data) could explain the stronger autotrophy Laas et al. [26] observed in Võrtsjärv compared to this study. GPP_phytoplankton_ might also have been underestimated in our study because under-ice production was not considered by the GPP_phytoplankton_ model we used [43]. However transparent ice conditions are uncommon in Võrtsjärv and very short-lived because of fast ice-break-up in spring. Consequently, the underestimation of GPP_phytoplankton_ is probably minimal. Our estimates of plankton biomass and primary production are highly reliable as these parameters were monitored *in situ* during several years.

Though heterotrophy is the norm for unproductive oligotrophic and dystrophic lakes [40], it is generally thought that eutrophic lakes like Võrtsjärv should be autotrophic. As the slope of the power relationship between R (y-axis) and GPP (x-axis) is lower than 1 [56], it leads to a smaller increase of R relative to GPP. However, Duarte and Agusti [56] considered that the required GPP for switching from net yearly heterotrophy to net autotrophy was 1 g O_2_ m^−3^ day^−1^, i.e. 468 mg C m^−3^ day^−1^ using their molar conversion factor of 1.25. Võrtsjärv, with an average GPP of 230 mg C m^−3^ day^−1^ does not reach this threshold and belongs thus to the presumably heterotrophic group of lakes.

The annual average primary production in Võrtsjärv is similar to the production of eutrophic hemiboreal lakes from Quebec (Lake Waterloo; [57]), Saskatchewan (Lake Katepwa; [58]) and Denmark (Frederiksborg Slotssø; [5]). According to our results, phytoplankton was the main primary producer in Võrtsjärv which is consistent with GPP patterns observed in eutrophic lakes [59]. However, on a whole-lake scale the system is heterotrophic. It means that planktonic and benthic consumers are dependent of the mineralization of not only autochthonous (dead phytoplankton and macrophytes) but also of allochthonous (tributaries) organic matter ([9], [60]). As previous research has demonstrated, the labile fraction of autochthonous dissolved organic carbon (DOC) is high in Võrtsjärv and constitutes a suitable substrate for bacteria growth [61]. Another influx of organic carbon can be provided by allochthonous material derived from the catchment [62]. Indeed, a recent mass-balance study showed that Võrtsjärv receives generally more DOC than it loses through the outflow, meaning that planktonic consumers process this DOC and turn the lake into a DOC sink [63].

Phytoplankton's own production to respiration ratio (GPP/R) was low (1.28). Two factors could explain this observation: firstly, the high turbidity of the lake (average Secchi depth <1 m) severely limits production, constraining the depth of the euphotic zone. Additionally, adverse light conditions favour shade-tolerant species. In case of Võrtsjärv, these species are slow growing cyanobacteria which have low photosynthetic efficiency compared to larger phytoplankton cells such as diatoms [37].

Macrophyte contribution to the whole-lake GPP, though far from negligible (10%) was much smaller than that observed in clear, benthos-dominated lakes. Andersson and Kumblad [11] reported a proportion of GPP (31%) made by macrophytes in a small (0.28 km^2^) oligotrophic Swedish lake that is very similar to the average value (29%) found by Vadeboncoeur et al. [59] in a literature review of 17, mostly small and oligotrophic lakes. Dominance of phytoplankton in Võrtsjärv primary production was more akin to conditions prevailing in turbid and shallow Lake Søbygård in Denmark [21]. We did not take into account epipelic primary production, although it has been reported to account up to 10% of the whole GPP in a turbid lake, but only during temporary clear-water conditions [21] which did not occur in Võrtsjärv. Light-limitation may also be the strongest factor explaining lower-than-expected macrophyte contribution to the metabolism of turbid lakes. Interestingly, the own respiration rates of macrophytes on an aerial basis were the highest among all functional groups considered in this study. Both GPP/R ratio (1.45) and the absolute R values we have calculated in *M. spicatum* were nevertheless in the same range with those reported by Middelburg et al. [64] for macrophyte beds of other species.

### Heterotrophic shift in metabolism caused by microbial loop consumers

The shift towards heterotrophy in midsummer was caused by an increased protozooplankton and bacterioplankton oxygen demand. The mean contribution of bacterioplankton to plankton respiration was indeed twice higher than that reported by Biddanda et al. [65] for lakes with a similar trophic state. The cause of this difference stems from the species composition of phytoplankton in turbid lakes. Phytoplankton in this type of lakes is often dominated by filamentous cyanobacteria which are not suitable food even for large-size zooplankton [66], creating a trophic bottleneck. Consequently the unfavourable feeding conditions have shaped the planktonic crustacean community towards dominance of less efficient small-sized cladocerans and cyclopoid copepods. Planktonic primary production is utilized by unicellular organisms such as planktonic bacteria, ciliates, and small rotifers ([67], [68]). These bacteria and protozoans are inefficient consumers with low assimilation rates ([30], [69]) and thus release large amounts of CO_2_ relative to their biomass. These findings illustrate the importance of considering food web structure differences for explaining metabolism gaps between lakes of similar trophic state.

### Low contribution of benthic consumers

The overall very low average contribution of benthic consumers (bacteria, ciliates, macroinvertebrates) to lake metabolism (3%) might appear surprising for a shallow lake where most of the recycling of organic matter is supposed to take place in the benthic zone [70]. This contribution is much lower to what is found in oligotrophic clear water lakes [11]. Besides a possible underestimation of benthic carbon demand, two factors can explain this discrepancy: benthic macroinvertebrates own metabolic levels are two orders of magnitude lower than those of unicellular organisms (50 and 4500 µL O_2_ g ww^−1^ h^−1^, respectively, [71]). Another factor can be the relatively low biomass of benthic consumers in Võrtsjärv compared to neighbouring lakes such as Lake Peipsi [54]. The small standing stock of zoobenthos is likely caused by the abundance of benthivorous fish which build up 70% of fish biomass [22] and 14% of its respiration in this lake (this study). We can thus conclude that the small contribution of benthic consumers to lake metabolism is a result of their low metabolic rates combined with top-down control by predatory fish which maintains low density of these organisms.

### Model validation

**Figure 6:**
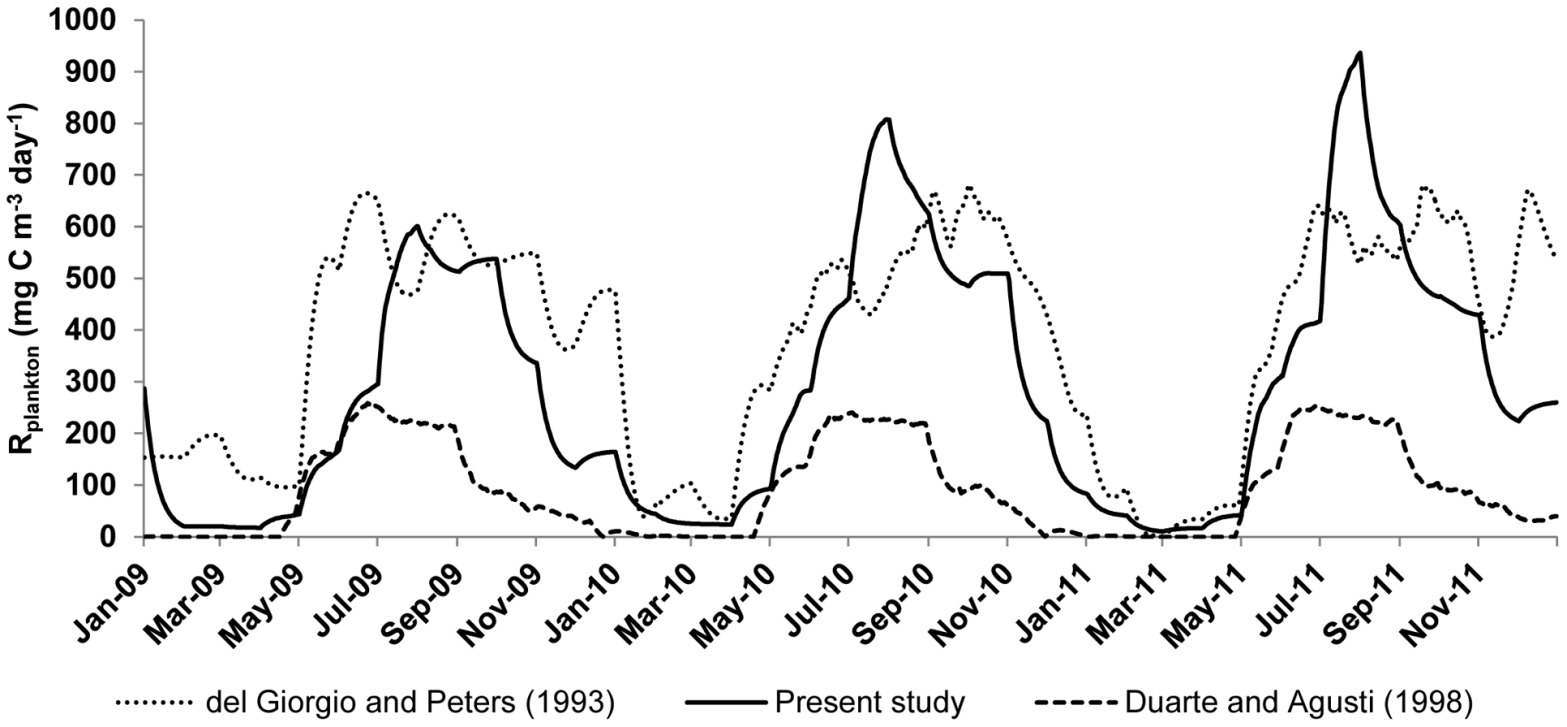
Comparison of plankton respiration (mg C m^−3^ day^−1^) in Võrtsjärv calculated with an ecosystem approach (this study), chl-*a*-based del Giorgio and Peters [40] and GPP-based Duarte and Agusti [56] equations. In this study R_plankton_  =  R_phytoplankton_ + R_bacterioplankton_ + R_protozooplankton_ + R_metazooplankton_.

Our ecosystem approach was validated by strong correlations found between the calculated GPP and R values and the already published *in situ* measurements of GPP and R [26] though only during the ice-free period. A comparison shows that our R values are within the same order of magnitude as those assessed with models by del Giorgio and Peters [40] and Duarte and Agusti ([56] Fig. 6) though generally higher. Higher R yielded by the ecosystem approach can be caused by the biomass peaks of microbial loop organisms observed in summer which are not tracked by the other models.

However, our approach is not free of uncertainties which can originate from functional group sampling, equations used and interpolation of the results to whole-lake surface and volume. Generally, one of the main weaknesses of the ecosystem approach for calculating the carbon budget is the large aggregation error associated with scaling of the values obtained at cell level to the whole ecosystem. In our study two factors minimize sampling and interpolation-related errors: firstly, the homogeneity of Võrtsjärv [12] which is remarkable for such a large lake, and, secondly, the relatively high sampling frequency and duration (monthly during three years) for most of functional groups, especially the planktonic ones. Equation-related errors are more challenging to assess though. Straight-from-literature respiration rates and allometric equations allow only rough estimates of actual respiration rates which are influenced by many more factors than cell size alone. Furthermore, for technical reasons respiration rates of unicellular organisms are extremely hard to assess in nature so that these estimates are often obtained from *in vitro* metapopulations instead [6]. Finally, there is no guarantee that the respiration rates we used and were measured in different systems, were appropriate for our study site conditions. However, it is difficult to estimate individual errors and their effect on cumulative propagated errors. Estimates of error distributions themselves might require bootstrap Monte Carlo techniques which may yield an unquantified uncertainty [10]. Yet, the most important value for each of the equations we have used is the functional group biomass, which for many taxa is spanning over several orders of magnitudes. This parameter is also the easiest to measure and the one that is the least subject to errors.

## Conclusion

To our knowledge, this is the first time when such a detailed metabolic balance based on functional group metabolic rates of a lake has been calculated for such a long time period (three years) and with a frequency high enough for noticing monthly trends. This method provided novel information about seasonal contribution of functional groups to the whole-lake metabolism. Of course, caution is needed when using output values resulting from allometric relationships, and relative contributions of individual functional group to the lake metabolic rates are probably more reliable than the absolute values. The ecosystem approach pointed out the high share of phytoplankton in the whole-lake GPP and R, the importance of microbial planktonic consumers in the metabolism, and also the low importance of benthic consumers in C emissions in shallow and turbid lakes. Furthermore, it appears that lake metabolism assessment benefits from a dynamic approach. Indeed, research conducted in highly productive summer period only would have concluded, inappropriately, net autotrophy of the system, though the dynamic approach showed a switching pattern. Ideally, the ecosystem approach can be combined with the diel O_2_ approach for investigating metabolism shifts caused by changes in the functional group dominance and food-web processes. Although some of the studied functional groups (both groups of fish) lacked a good temporal resolution, our approach provided more detail for the plankton components which comprise the metabolically most active parts of eutrophic lakes. On the other hand, obtaining such a resolution requires a large amount of information for each component to be taken into account. The ecosystem approach accuracy is thus fully dependent of the degree of knowledge available about lake functional groups.

## Supporting Information

Table S1 **Parameter database for functional group metabolic measurements.**
(XLSX)

Table S2 **Composition of benthic and planktonic communities in the 2009–2011 period.**
(XLSX)
